# Diabetes technology and sexual health: which role?

**DOI:** 10.1007/s40618-023-02237-7

**Published:** 2023-11-21

**Authors:** V. Zamponi, J. Haxhi, G. Pugliese, A. Faggiano, R. Mazzilli

**Affiliations:** Endocrine-Metabolic Unit, Department of Clinical and Molecular Medicine, Sapienza University of Rome, Sant’ Andrea Hospital, via di Grottarossa, 1035-1039 Rome, Italy

**Keywords:** Diabetes, Technology, Female sexual dysfunction, Continuous glucose monitoring, Continuous subcutaneous insulin infusion, Erectile dysfunction

## Abstract

**Purpose:**

The aim of this review is to evaluate the effects of new technology used in the management of diabetes mellitus (DM), including the use of continuous glucose monitoring (CGM) and the administration of insulin through continuous subcutaneous insulin infusion (CSII), on male and female sexual function.

**Methods:**

This narrative review was performed for all available prospective, retrospective and review articles, published up to June 2023 in PubMed. Data were extracted from the text and from the tables of the manuscript.

**Results:**

Sexual dysfunctions are an underestimated comorbidity of DM in both male and female. Although erectile dysfunction (ED) is recognized by the guidelines as a complication of DM, female sexual dysfunction (FSD) is poorly investigated in clinical setting. In addition to the complications of DM, the different types of therapies can also influence male and female sexual response. Furthermore, insulin therapy can be administered through multiple-daily injections (MDI) or a CSII. The new technologies in the field of DM allow better glycemic control which results in a reduction in the occurrence or aggravation of complications of DM. Despite this evidence, few data are available on the impact of new technologies on sexual dysfunctions.

**Conclusions:**

The use of DM technology might affect sexual function due to the risk of a worse body image, as well as discomfort related to CSII disconnection during sexual activity. However, the use is related to an improved metabolic control, which, in the long-term associates to a reduction in all diabetes complications, including sexual function.

## Introduction

Diabetes mellitus (DM) is a group of metabolic diseases with a high prevalence in general population, characterized by hyperglycemia resulting from defects in insulin secretion, insulin action, or both. The most prevalent form is represented by type 2 diabetes (T2DM), while in about 5–10% of cases type 1 diabetes (T1DM) could occur. Long-term complications of DM include atherosclerotic cardiovascular, peripheral arterial, and cerebrovascular disease, retinopathy, nephropathy, peripheral neuropathy and autonomic neuropathy, causing gastrointestinal, genitourinary, and cardiovascular symptoms. A further complication, caused by both neurological and vascular effects, is sexual dysfunction [1].

The World Health Organization (WHO) defines sexual health as encompassing physical, emotional, psychological, and social well-being, emphasizing sexual desire and fulfillment, rather than solely the absence of disease, dysfunction, or disability. Sexual health is determined by the interplay of cardiovascular, neurological, and hormonal elements, and it can be influenced by individual factors and interpersonal relationships. It is well known that DM and sexual dysfunction are strictly related. DM can contribute to sexual dysfunction through various mechanisms, including endothelial damage, neuropathy, hormonal imbalances, and psychological factors. All of these factors could induce a negative impact on quality of life (QoL) [2, 3]. Although new technologies have become a must-have instrument for people with T1DM, it is increasingly being recognized that people with T2DM can benefit from technology to achieve recommended glycemic control [4].

Recent evidence highlights the beneficial impact of anti-hyperglycemic drugs on male and female sexual function [5, 6]. However, little is known regarding the effect of insulin treatment, the type of administration and new technology used in the management of DM, including the use of continuous glucose monitoring (CGM) and insulin therapy through continuous subcutaneous insulin infusion (CSII).

The aim of this review is to evaluate the effects of new technology used in the management of DM, both CGM and CSII, on male and female sexual function.

## Methods

This narrative review was performed for all available prospective, retrospective and review articles, published up to July 2023 in PubMed. Data were extracted from the text and from the tables of the manuscript. All studies reporting any measure of sexual function were reviewed.

**Fig. 1:**
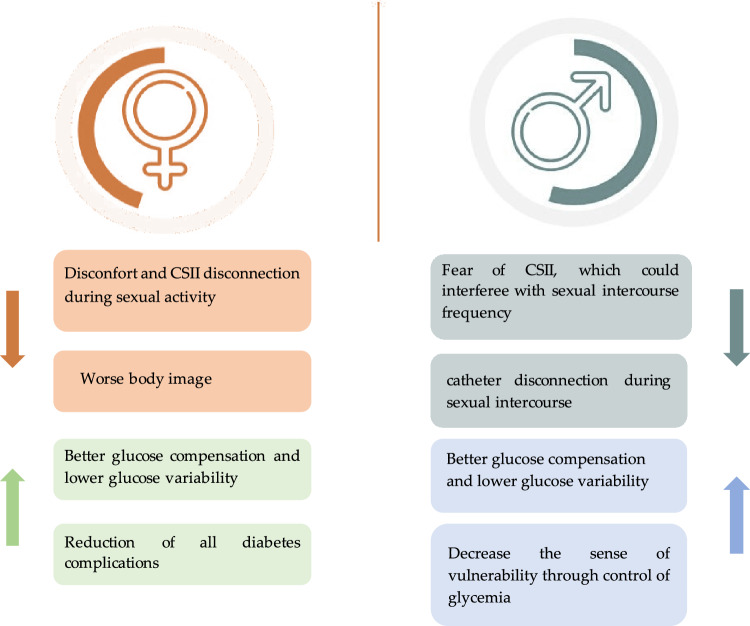
Pros and cons of continuous subcutaneous insulin infusion (CSII) and continuous glucose monitoring (CGM) use on male and female sexual function

The keyword search used included “diabetes technology and female sexual function”, “continuous glucose monitoring (CGM) and female sexual function”, “continuous subcutaneous insulin infusion (CSII) and female sexual function”, “diabetes technology and male sexual function”, “continuous glucose monitoring (CGM) and male sexual function”, “continuous subcutaneous insulin infusion (CSII) and male sexual function”, “diabetes technology and erectile dysfunction”, “continuous glucose monitoring (CGM) and erectile dysfunction”, “continuous subcutaneous insulin infusion (CSII) and erectile dysfunction”, “diabetes technology and premature ejaculation”, “continuous glucose monitoring (CGM) and premature ejaculation”, “continuous subcutaneous insulin infusion (CSII) and premature ejaculation”, “diabetes technology and hypogonadism”, “continuous glucose monitoring (CGM) and hypogonadism”, “continuous subcutaneous insulin infusion (CSII) and hypogonadism” (Fig. 1).

### Technology and diabetes

T1DM and severe form of T2DM are characterized by lack of insulin production from pancreatic beta cells which, without the proper treatment, could lead to potentially lethal complications due to severe hyperglycemia. Insulin therapy, either through multiple-daily injections (MDI) or through an insulin pump, is crucial to prevent acute and chronic complications. Both therapies, need to be associated to glucose monitoring, which is used to guide decision-making on insulin dose and to get feedback after insulin has been administered. The number of times-per-day a patient measures blood glucose, correlates with glycemic control [7]. However, self-monitoring blood glucose (SMBG) provides static and point-in-time glucose measurements [8]. CGM on the other hand, provides frequent measurements (every 2–5 min) of glucose concentrations in the interstitial fluid, which allows to appreciate more dynamic glucose parameters such as post-prandial glycemia, overall glucose variability and asymptomatic episodes of hypoglycemia, which would otherwise be missed [8]. Also, the ambulatory glucose profile (AGP) derived from CGM data gives the possibility to evaluate overall glycemic control in a standardized manner and permits between-patients comparison [9]. Using a real-time CGM, significantly reduces the risk of severe hypoglycemia, ketoacidosis, and reduces glycemic variability, improving overall glycemic control [10, 11]. Miller and colleagues demonstrated that the earlier CGM is used after diagnosis, the better the glycemic control achieved, with those starting CGM use at diabetes onset having the most benefit, both short-term and long-term [12]. Recent CGM systems have improved accuracy, and many do not need calibration with capillary glucose values. Some of them can now be used as a substitute of capillary measurements in insulin dosing and can be used by the algorithms guiding insulin delivery in hybrid closed-loop systems (HCL). Closed-loop insulin delivery has showed superior results in glycemic control compared to sensor-augmented pump (SAP) therapy [13].

As demonstrated by the DCCT-EDIC trial, good glycemic control is essential in preventing long-term diabetes complications [14]. High HbA1c and low time in range (TIR) are associated to a higher risk of micro- and macro-vascular complications [15].

### Impact of technology on male sexual function

Erectile dysfunction (ED) and premature ejaculation (PE) are the two most prevalent sexual dysfunctions in male [16]. ED is the inability to achieve or maintain sufficient penile erection to obtain satisfactory sexual activity [17], while PE is the inability to control or delay ejaculation, both of which can result in dissatisfaction or distress for the couple [18]. Often, PE is a consequence of poor erection [19]. Both ED and PE have been associated with hypogonadism, DM [19] and with a poor quality of life of patients and their partners [21, 22].

#### Erectile dysfunction

Glycemic control is an important parameter to assess, due to the well-established negative impact on sexual function [22, 23]. The prevalence of ED in patients with DM ranges from 26 to 66% [2].

On the other hand, recent evidence highlighted that antidiabetic agents, with their different mechanisms of action, could have significant direct and indirect effects on sexuality [5, 25–27]. However, to date, few studies have assessed the effect of insulin treatment in men with ED and even fewer have specifically focused on CSII. Maiorino et al., evaluated sexual function in subjects with T1DM, aged 18–35 years, and did not observe any differences with respect to insulin regimens (MDI or CSII) [28].

Conversely, Kesavadev et al. compared CSII and MDI therapies, and reported a significant reduction in ED severity and an increase in IIEF-5 scores in patients with T2DM using CSII [29]. The authors also performed an evaluation through a professional continuous glucose monitoring (P-CGM) at baseline and after 3 months and observed a reduction in glycemic variability in the CSII group which could have contributed to the improved outcomes. These results are encouraging and could improve acceptance of pump therapy even in those patients who refuse such therapy because they fear it might worsen with their sex lives and reduce intercourse frequency.

Interestingly, Riveline et al. used a questionnaire on inconvenience/convenience of the pump and catheter to assess the impact of CSII therapy on sexual activity in men and women with DM, through a. In both, men and women, the response to the question “Does the pump have an influence on your sexual activity?”, was “no” in 90% and “yes” in the remaining 10%. On multivariate analyses, male sex was independently associated with catheter disconnection during sexual intercourse [30].

Finally, Robertson et al. analyzed the effects of diabetes technology on anxiety, body image, and sexual activity in people with T1D who adopt externally worn (CSII and CGM) [31]. The authors did not observe differences in anxiety, body image concerns and in frequency of sexual intercourse. Furthermore, sexual satisfaction appeared to not be affected by technology. Particularly, CSII users reported developing a pragmatic solution, namely disconnecting the insulin pump to overcome the problems during sexual intercourse. Similar conclusions were reported by Garza et al. [32], who performed the “Perceptions, Ideas, Reflections and Expectations (INSPIRE) study” and found that the use of CSII may decrease the sense of vulnerability through provision of greater control of glycemia, also minimizing discomforts in the context of sexual intimacy.

#### Premature ejaculation

Few data are available on the impact of technology on PE. A study conducted by Bellastella et al., evaluated the prevalence of PE and the influence of glycemic control on ejaculatory function in 100 males with T1DM.

PE was assessed with the premature ejaculation diagnostic tool (PEDT) and the self-estimated intravaginal ejaculatory latency time (IELT). Glucose variability was evaluated by CGM for a 7-day period with a DexCom G4 CGM system. They found an overall PE prevalence of 24%. The prevalence was similar to that described in the non-diabetic population, which is approximately 19%-t 30% [33]. A higher PEDT score was associated with higher levels of low blood glucose indices (LBGI) (*r* = 0.43; *p* = 0.01), but not with higher standard deviations of blood glucose (BGSD) (*r* = 0.1, *p* = 0.6), higher mean amplitude of glycemic excursions (MAGEs) (*r* =  − 0.1; *p* = 0.4), or higher levels of high blood glucose indices (HBGI) (*r* = 0.1; *p* = 0.6).

The authors suggest that hypoglycemia-induced activation of the adrenergic system or inhibition of serotoninergic neuronal activity, which are both associated with a reduction of ejaculation time, might explain the association of hypoglycemia with PE [34, 35].

#### Hypogonadism

Considering male hypogonadism, several cross‐sectional studies reported up to 40% of men with T2DM exhibit low testosterone levels [2]. A recent study conducted by Defeudis et al. [26], evaluated the effect of testosterone replacement therapy (TRT) on glycemic control and variability measured with aCGM, in people with T2DM and ED. The authors found no significant differences in TIR, time above range (TAR, > 140 mg/dL), time below range (TBR, < 70 mg/dL), estimated HbA1c, area under the curve (AUC) for blood glucose values above and below the target during the intervention period. A previous study conducted by Ding et al. speculated that increasing testosterone levels in hypogonadal men with DM could lead to an increase in glycemic variability after TRT [36], but the analysis of data from a the CGM highlighted no significant deterioration of any parameters.

In this context, CGM was a useful tool to confirm the glycometabolic safety of the TRT, even in terms of the most novel standardized glycemic metrics.

### Impact of technology on female sexual function

In the Diagnostic and Statistical Manual of Mental Disorders, 5th edition (DSM-5), female sexual dysfunction (FSD) refers to a set of persistent and recurrent difficulties or problems experienced by women that involve one or more stages of sexual response. The diagnosis of FSD requires the presence of clinically significant distress or interpersonal difficulties associated with these sexual difficulties [37].

The reliable prevalence of FSD in the general population is still unclear. Indeed, studies report very wide prevalence variability ranging from 20% in pre-menopausal age to 60% after menopause [38, 39].

There is a recognized relationship between DM and sexual dysfunction. DM can contribute to sexual dysfunction through various mechanisms, including the effects of high blood sugar levels, endothelial damage, neuropathy, hormonal imbalances and psychological factors. All these factors have a negative impact on QoL [2, 3].

Although the etiopathogenic mechanisms of DM underlying sexual dysfunction are the same for both genders, only ED is recognized by the guidelines as a complication of DM, leading to underestimation of possible female sexual problems in the clinical setting [40].

Several studies investigated the impact of DM on female sexual health, demonstrating that the prevalence of FSD in DM population is high, ranging from 20 to 51% for T1DM and from 17 to 68.6% for T2DM [3, 41–43].

Various cardio-metabolic risk factors, including atherogenic dyslipidemia, the presence of metabolic syndrome and hyperglycemia are associated with compromised sexual arousal. This phase of sexual response seems to be the most compromised in women with DM, as previously demonstrated for men with DM [44].

Limited research has been conducted to assess the impact of hypoglycemic medications on the sexual well-being of women with diabetes [25]. The treatment approach for T1DM typically involves basal-bolus insulin therapy, which can be administered through MDI or CSII.

Several studies have been conducted on the impact of new diabetes technologies on sexuality. In these studies, great discomfort emerges from users of CSII and CGM during sexual activity that lead to the disconnection of insulin pump in about 75% of cases. The main limitations that are expressed by CSII and CGM users are the negative impact on their body image which does not allow to establish good intimacy with the partner [32, 45, 46], even if it appears difficult to understand why a removable sensor could have such a detrimental effect. In this regard, no studies are available to clearly understand if the beneficial psychological effect outweigh the detrimental ones.

In contrast, Reveline and colleagues highlighted that only 10% of the diabetic patients treated with CSII therapy reported a negative impact on their sexual life. The authors conducted a multivariate analysis, highlighting that the disconnection of the catheter during sexual intercourse was independently associated with male gender, younger age, higher HbA1c levels, and catheter discomfort [31].

The major limitations of these studies are that they did not perform gender-differentiated statistical analyzes and that validated questionnaires investigating FSDs were not used.

In the study of Maiorino et al. (METRO study) on young T1DM female patients, a specific analysis on the impact of CSII on female sexual health was conducted. Sexual function was assessed by validated questionnaires such as Female Sexual Function Index 19 (FSFI) and the Female Sexual Distress Scale (FSDS). The authors highlighted that the prevalence of FSD was significantly lower in diabetic women on CSII compared to women on MDI (*p* = 0.035). Diabetic women utilizing MDI demonstrated not only a decreased mean total score of the FSFI (*p* = 0.039), but also a lower score in the arousal (*p* = 0.024) and satisfaction domains (*p* = 0.016), compared to the CSII group. Furthermore, the MDI group presented a higher sexual distress score compared to CSII users [47].

These results were confirmed by another Italian case–control study on T1DM female patients, suggesting that CSII does not appear to be a real impediment to female sexual well-being [3].

Additionally, in both studies, FSFI score, and its single items showed a negative association with HbA1c levels and the presence of diabetic complications [3, 47]. This association may be attributed to a reduced glucose variability among patients using CSII compared to MDI, as suggested by Longo et al. [47]. Indeed, in this study, the prevalence of FSD was significantly higher in women with high glucose variability compared to women with low glucose variability (*p* = 0.008). Furthermore, the FSFI total score (*p* = 0.002) and scores in the domains of arousal (*p* = 0.008) and pain (*p* = 0.022) were lower in patients with higher glucose variability, confirming that not only the chronic exposure of high blood sugar but also its fluctuations could be predictive factors for the development of sexual dysfunction in both male and female [48].

### Conclusions

In conclusion, the use of diabetes technology might affect sexual function due to the risk of a worse body image, as well as discomfort related to CSII disconnection during sexual activity. However, the use of CSII and CGM is related to an improved metabolic control, which, in the long-term associates to a reduction in all diabetes complications, mainly cardiovascular and neurological which are strictly related to the onset of sexual dysfunctions in both male and female. A well-controlled and non-complicated diabetes results in better sexual function. In addition, CGM and similar technologies are less “intrusive” than the prior methods of glucose monitoring and this can result in a better psychological status.

Existing evidence is insufficient to evaluate gender differences, and further studies, with larger sample sizes, are necessary to confirm the impact of technology on sexual function in men and women with diabetes.
